# COVID-19: do it like Greece. Why Greece is coping with COVID-19 better than other countries?

**DOI:** 10.11604/pamj.supp.2020.35.2.24946

**Published:** 2020-07-13

**Authors:** Christos Damaskos, Anna Garmpi, Vasiliki Epameinondas Georgakopoulou, Paraskevi Farmaki, Evangelos Diamantis, Dimitrios Dimitroulis, Serena Valsami, Konstantinos Kontzoglou, Efstathios Antonios Antoniou, Zoi Damaskou, Lampros Nikolidakis, Athanasios Syllaios, Georgios Marinos, Nikolaos Trakas, Nikolaos Garmpis

**Affiliations:** 1 Renal Transplantation Unit, Laiko General Hospital, Athens, Greece,; 2 N.S. Christeas Laboratory of Experimental Surgery and Surgical Research, Medical School, National and Kapodistrian University of Athens, Athens, Greece,; 3 First Department of Propedeutic Internal Medicine, Laiko General Hospital, Medical School, National and Kapodistrian University of Athens, Athens, Greece,; 4 Department of Pulmonology, Laiko General Hospital, Athens, Greece,; 5 First Department of Pulmonology, Sismanogleio Hospital, Athens, Greece,; 6 First Department of Pediatrics, Agia Sofia Children´s Hospital, Medical School, National and Kapodistrian University of Athens, Athens, Greece,; 7 Department of Endocrinology and Diabetes Center, G. Gennimatas General Hospital, Athens, Greece,; 8 Second Department of Propedeutic Surgery, Laiko General Hospital, Medical School, National and Kapodistrian University of Athens, Athens, Greece,; 9 sssDepartment of Hematology Laboratory Blood Bank, Aretaieion Hospital, National and Kapodistrian University of Athens Medical School, Greece,; 10 Department of Internal Medicine, “Ygeias Melathron” Hospital, Athens, Greece,; 11 First Department of Surgery, Laiko General Hospital, Medical School, National and Kapodistrian University of Athens, Athens, Greece,; 12 Department of Emergency Medicine, Laiko General Hospital, Athens, Greece,; 13 Department of Biochemistry, Sismanogleio Hospital, Athens, Greece.

**Keywords:** COVID-19, pandemic, public health, Greece

## To the Editors of the Pan African Medical Journal

Several European countries have been badly affected by the novel coronavirus pandemic, but Greece reacted early and decisively, and it seems to be working. However, the country´s ability to cope with a public health emergency of such proportions was far away from considered as something given. The main reason was the debt financial crisis which took place during the last decade and actually plunged the country; economists and analysts argue that the economy was contracted up to 26%. Taking this into account, it is easily assumed that Greece´s public health system actually bore the brunt. According to official data from the ministry of health, at the beginning of pandemic, Greek hospitals had only 560 intensive care unit (ICU) beds. In addition, the geomorphological structure of the country itself with several little islands was an additional “disadvantage” as most of them had only primary care units making it therefore necessary for potential future patients to be transported to the mainland in order to be treated effectively. All these objective weaknesses and the fact that Greece had a large elderly population, left no room for time to waste or embracement of strategies like acquiring herd immunity. The Greek government took a relatively hard decision: prioritize science over politics in a country that had just started to show the first signs of financial recovery. When the first patients were announced in the nearby Italy, every citizen that had travelled to Italy or China and had fever or cough was examined and isolated. Subsequently, the appearance of the first confirmed case in late February, triggered a waterfall of measures.

Following the confirmation of the first three cases in Greece, on 27th of February all carnival events in the country were cancelled, before the first death. On 10th of March, with 89 confirmed cases and no deaths in the country, the government decided to suspend the operation of educational institutions of all levels nationwide, although young children and adolescents were not regarded as a “high risk group”. They were carriers of the virus and therefore increased the risk of transmitted it to the elderly. It is crucial at that point to underline that the rest European countries took that decision only several weeks later probably giving the virus a significant lead, difficult to be reversed. The first death from COVID-19 in Greece was announced on 12 March. From 13th to 16th of March all possible meeting points like cafes, restaurants, nightclubs, gyms, malls, cinemas, retail stores, museums and archaeological sites were also shut down and all churches of any religion or dogma were suspended. On 18th and 19th of March, the government announced a series of measures of more than 10 billion euros to support the economy, businesses and employees. However, all these were not generally accepted without complaints and the government was forced to take “extreme” measures like banning all public gatherings of more than 10 people.

On 22nd of March, the Greek government announced restrictions on all non-necessary movement throughout the country. Since that date, movement outside the house was permitted only for seven categories of reasons: 1. moving to or from one’s workplace and only during working hours, 2. going to the pharmacy or visiting a doctor 3. going to stores selling food, 4. going to the bank only for services not possible online, 5. assisting a person needing help 6. going to a major ritual (funeral, marriage, baptism) or movement, for divorced parents, which is essential for contact with their children, and 7. moving outdoors for personal exercising or taking one’s pet out for a walk. Citizens leaving their homes are required to carry their police ID or passport, as well as a signed attestation in which the purpose or category of travel is stated. The Hellenic Police, the Municipal Police, the Hellenic Coast Guard and the National Transparency Authority were empowered to enforce the restrictions and could issue fines for each offence. These restrictions were extended until 4th of May. Additionally, from 23rd of March, Greece suspended all passenger flights to and from countries with high rates of transmission as well as sea, rail and road connections, with an exception for Greek citizens and those who have residence permits or whose main residence is in Greece, as well as trucks and ships transporting goods.

**Figure 1:**
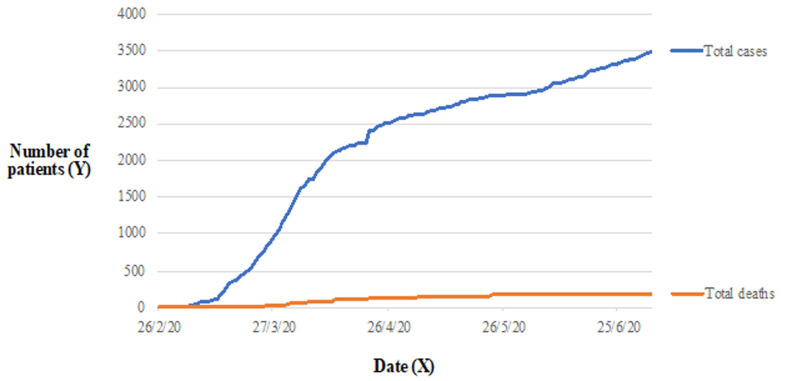
graph showing the amount of total confirmed cases and total deaths due to COVID-19

The measures put in place in Greece are among the most proactive and strict in Europe and have been credited internationally for having slowed the spread of the disease and having kept the number of deaths among the lowest in Europe, earlier from many other countries. Moreover, in an effort to modernize the notorious Greek bureaucracy, the vast majority of transactions with the banks or public services is now done electronically; even medical prescriptions are sent via phone messages. Greece obtained millions of masks both for health professionals and for patients. More importantly, thanks to the donations of several Greeks and the partnership of the private sector, Greece managed to double the available ICUs beds. There are now 1017 ICUs beds, including the ones from private clinics. In the span of only a few weeks 3748 health personnel were recruited. High-dependency units were also added to hospitals; these are areas usually located close to the intensive care unit, where patients can be treated more extensively than on a normal ward, but not to the point of intensive care. The National Public Health Organization had set up telephone lines for the citizens to be informed about the coronavirus, and to be consulted in case they have symptoms compatible with COVID-19 infection. Several therapeutic strategies have been suggested and applied by Greek doctors for different groups of patients [1-4]. At that point, we need to underline the exemplary attitude of the unruly Greeks which complied with all these changes in their daily routine and thy stayed home. Fortunately, the results were far better than expected and now everybody is talking about the flattening of the curve which will eventually allow Greece to gradually exit the lock-down (Figure 1) [5].

From June 1st, the year-round hotels, campsites and camps were reopened, while from June 15th and onwards, the seasonal hotels were also allowed to welcome visitors from abroad. Flights to Eleftherios Venizelos Athens International Airport restarted from June 15th, but only from countries with good epidemiological criteria. From July 1st flights will restart in all of country´s airports. There is a government´s plan including in detail the rules of hygiene and protection for the operation of tourist accommodation, for tourist buses, for car rental companies, for maritime transport with passenger and passenger-vehicle ferries, air transport, where rules of hygiene are defined within the aircraft at the airport, as well as controls at the entrance gates with questionnaires, sampling tests and specific procedures. Sampling and inspections will be both thorough and rigorous, where necessary, in tourist arrivals, in order to keep Greek citizens safe. Greek government is going to proceed to the mandatory definition of a collaborating doctor with each accommodation, as the first evaluation point. Respectively, a coordinator from the accommodation is appointed to manage suspected cases. The health system is going to be ready at any time to transport a patient to a health facility, if necessary, from the island with coverage from nearby destinations with transport time less than two hours. Health care professionals were at first anxious due to the healthcare system weakened by the previous crisis. In Italy, for example, we were informed that a lot of people were affected in a short time, that the health system didn´t have the flexibility to deal with the excess cases and that there were doctors and nurses infected from the virus and eventually died. These dangers were not far away from the reality in Greece, but thanks to the quick reaction of the Greek government, there was health stuff recruitment, increase in intensive care beds, increase in available RT-PCR test and protective equipment. Now, we feel confidence in case we have a second wave of COVID-19 in Greece.
